# Microstructure and Thermophysical Characterization of Tetra-Arsenic Biselenide As_4_Se_2_ Alloy Nanostructured by Mechanical Milling

**DOI:** 10.3390/ma18112422

**Published:** 2025-05-22

**Authors:** Oleh Shpotyuk, Andrzej Kozdras, Yaroslav Shpotyuk, Guang Yang, Zdenka Lukáčová Bujňáková

**Affiliations:** 1 Faculty of Mathematics and Natural Sciences, Jan Dlugosz University in Czestochowa, 13/15, al. Armii Krajowej, 42-200 Czestochowa, Poland; 2 O.G. Vlokh Institute of Physical Optics, Ivan Franco National University of Lviv, 23, Dragomanov Str., 79005 Lviv, Ukraine; 3 Institute of Physics, Opole University of Technology, 75, Ozimska Str., 45-370 Opole, Poland; a.kozdras@po.opole.pl; 4 Institute of Physics, University of Rzeszow, 1, Pigonia Str., 35-959 Rzeszow, Poland; yashpotyuk@gmail.com; 5 Department of Sensor and Semiconductor Electronics, Ivan Franko National University of Lviv, 107 Tarnavskoho Str., 79017 Lviv, Ukraine; 6 School of Materials Science and Engineering, Shanghai University, Shanghai 200444, China; guangyang@shu.edu.cn; 7 Department of Mechanochemistry, Institute of Geotechnics of Slovak Academy of Sciences, 45, Watsonova Str., 04001 Košice, Slovakia; bujnakova@saske.sk

**Keywords:** nanostructurization, amorphous alloy, arsenoselenide, polyamorphic transition, nanomilling, heat-transfer phenomena

## Abstract

Nanomilling-driven effects on polyamorphic transitions are examined in tetra-arsenic biselenide As_4_Se_2_ alloy, which is at the boundary of the glass-forming region in the As-Se system, using multifrequency temperature-modulated DSC-TOPEM^®^ technique, supported by X-ray powder diffraction (XRPD) and micro-Raman spectroscopy analysis. As shown by XRPD analysis, this alloy reveals a glassy–crystalline nature due to rhombohedral As and cubic As_2_O_3_ (arsenolite) inclusions, which especially grew after milling in a PVP (polyvinylpyrrolidone) water solution. At the medium-range structure level, nanomilling-driven changes are revealed as the disruption of intermediate-range ordering and enhancement of extended-range ordering. The generalized molecular-to-network amorphization trend in this alloy is confirmed by the microstructure response revealed in the broadened and obscured features in micro-Raman scattering spectra collected for nanomilled specimens. Thermophysical heat-transfer phenomena are defined by molecular-to-network polyamorphic transformations activated under nanomilling. The domination of thioarsenide-type As_4_Se_n_ entities in this alloy results in an abnormous nanomilling-driven network-enhanced glass transition temperature increase. The nanomilled alloys become notably stressed owing to the destruction of molecular thioarsenide and incorporation of their remnants into the newly polymerized arsenoselenide network. This effect is more pronounced in As_4_Se_2_ alloy subjected to dry nanomilling, while it is partly counterbalanced when this alloy is additionally subjected to wet milling in a PVP water solution, accompanied by the stabilization of the As_4_Se_2_/PVP nanocomposite.

## 1. Introduction

Nanostructured materials possessing a great variety of size-dependent phenomena attract great interest in the contemporary nanomaterials science community because of their very important and unique multi-functionality [1,2]. Being in a nanostructured state approaching extremely low nanometer and sub-nanometer length scales, such nanoscopic materials demonstrate an unprecedented tendency for multiphase occurrence, bringing about essential modifications to thermophysical heat-transfer responses to external influences [3,4,5,6,7]. Typically, a pronounced decreasing trend is observed in the characteristic interphase transition temperatures of multiphase nanomaterials below certain critical sizes, at which one metastable phase becomes thermodynamically more favorable over competing phases [4,5,6,7].

The nanostructured over-stoichiometric arsenoselenide alloys of the canonical As*_x_*Se_100−*x*_ system (*x* > 40) possessing molecular-network conformations based on thioarsenide-type As_4_Se_n_ entities (*n* = 0–6) [8] can be mentioned as typical examples of such substances widely used in contemporary chalcogenide photonics, optoelectronics, optics, telecommunication techniques, chemical sensing (biosensing), and other biomedical applications [9]. Being nanostructured under some external alterations like high-energy mechanical ball milling (also termed nanomilling), these materials attain a principally novel functionality (including improved bioavailability in a variety of arsenical drugs [10]), which is inaccessible in conventional technologies tailoring As-bearing compound alloys by quenching them from high-entropy melt [11,12].

The aim of this research is to conduct a comprehensive analysis of mechanoactivated thermophysical heat-transfer phenomena in one of such multi-nano-phase substances exemplified by arsenoselenide As_67_Se_33_ alloy, which is just at the boundary of the glass-forming region in the binary As-Se system [8], compositionally equivalent to tetra-arsenic biselenide (As_4_Se_2_ in the thioarsenide nomenclature accepted for molecular packing and electron density distribution in the iso-typical As-S system [13,14,15]), utilizing the advanced multifrequency temperature-modulated DSC-TOPEM^®^ technique alongside complementary microstructural analysis of the examined specimens by X-ray powder diffraction (XRPD) and micro-Raman scattering (micro-RS) spectroscopy analysis.

## 2. Materials and Methods

### 2.1. As_4_Se_2_ Alloy Fabrication, Mechanochemical Processing, and Preliminary Characterization

The As_67_Se_33_ alloy (compositionally equivalent to tetra-arsenic biselenide, As_4_Se_2_) was prepared using the elemental As and Se precursors of 5N purity acquired from Sigma Aldrich (St. Louis, MO, USA) and employing vibrational melt-quenching as described elsewhere [11,12]. Within this technological route, a sealed ampoule filled with As and Se was placed in a rocking furnace, heated to 925 K, and homogenized for 10 h. Then, it was cooled to ~775 K and quenched in water. To eliminate possible residual stress in the ingot under too rapid cooling, the ampoule was annealed for 1 h at ~400 K.

The alloy extracted from the ampoule was glassy–crystalline, as shown in preliminary XRPD analysis [12]. Indeed, the XRPD patterns collected for coarse-grained pieces of this alloy show diffuse peak halos ascribed to the ‘amorphous’ structure typical for glassy arsenoselenides supplemented by sharp ‘crystalline’ peaks originating from elemental As and its oxide As_2_O_3_ (arsenolite) [16,17,18]. In the as-fabricated melt-quenched As_4_Se_2_ specimen, the As phase prevails over As_2_O_3_ due to sharp ‘nanocrystalline’ reflexes superimposed on relatively weak and broad ‘amorphous’ halos. The density of the ingot was *ρ* = 4.450 g·cm^−3^ (as determined by Archimedes’ displacement method), and the glass transition temperature in mid-onset determination was *T*g = 120 °C (as derived from quick DSC scans at 10 K/min). These values correlate well with other counterparts in the As-Se system [8,9]. The mean interatomic spacing, d_sm_, calculated from the macroscopic density of this alloy was 3.79 Å.

The melt-quenching-derived As_4_Se_2_ alloy was subjected to mechanomilling in the Ar atmosphere at 500 min^−1^ speed for 60 min (using a Pulverisette 6 mill). Nanomilling was performed in a 250 mL WC chamber with 50 balls of 10 mm in diameter using ~3 g of the initial alloy sieved under 200 μm. Following milling, the powder was subjected to compression within a stainless-steel die under a pressure of approximately 0.7 GPa, yielding disc-shaped pellets (~6 mm in diameter and ~1 mm in thickness). This specific geometry was chosen for its optimal suitability in subsequent calorimetric investigations. In the XRPD pattern of this dry-nanomilled specimen, the diffuse peak halos prevail over broadened ‘nanocrystalline’ peaks from As and As_2_O_3_ phases, confirming the preference for the reamorphization transition between initial and final amorphous states [12]. The arrangement of diffuse peak halos in the XRPD pattern of the dry-nanomilled specimen was fit by decaying in their intensities, meaning that residual stress generated under mechanical grinding does not completely relax over the dry-nanopowdered substance.

Part of the preliminary dry-nanomilled alloy was additionally subjected to wet nanomilling in 300 mL of a 0.5% PVP (polyvinylpyrrolidone) water solution using a MiniCer mill (Netzsch, Selb, Germany). PVP of 40,000 g·mol^−1^ weight purchased from Sigma-Aldrich Co., LLC (St. Louis, MO, USA) was used. This 90 min processing was performed at 3500 min^−1^ speed under 85% loading of the milling shaft with yttrium-stabilized ZrO_2_ balls. After wet milling, the nanosuspension was dried at 70 °C and pelletized under ~0.7 GPa, producing a PVP-capped As_4_Se_2_ nanocomposite. In this As_4_Se_2_/PVP nanocomposite, the ‘crystalline’ lines of the As_2_O_3_ phase grow in intensity and become broader than those of the elemental As phase, and the arrangement of diffuse peak halos ascribed to the amorphous phase attains a character of irregularity for bulky glassy arsenoselenides [12]. Similar pellets were also fabricated from the PVP solution subjected to nanomilling to serve as a reference in calorimetric measurements for wet-nanomilled As_4_Se_2_ specimens.

### 2.2. Methodological Specificity of Microstructure and Thermophysical Characterization of Nanostructured Arsenoselenides

XRPD analysis (using a STOE STADI P diffractometer, Darmstadt, Germany) was performed in transmission mode with Cu Kα1 radiation to determine the phase composition and medium-range structure of the melt-quenched and nanostructured As_4_Se_2_ alloys.

Based on the collected XRPD patterns, phase composition was recognized using the data for arsenoselenide polymorphs, including the rhombohedral α-As phase (the space group *R*$\bar{3}$*m*, JCPDS card No. 72-1048 [17,18]) and cubic arsenolite As_2_O_3_ phase (the space group *Fd*3*m*, JCPDS card No. 36-1490 [18]). The amorphous nature of these alloys was confirmed by peak halos in their XRPD patterns (showing a characteristic three-peak structure [12,19,20,21]). These included the first sharp diffraction peak (FSDP) near the scattering vector *Q*_1~_(1–1.5) Å^−1^, as a signature of structural entities forming intermediate-range ordering (IRO) [19], the second sharp diffraction peak (SSDP) [21], and the principal diffraction peak (PDP) [21] near *Q*_2_~(1.8–2.2) Å^−1^, which was accepted as a signature of extended-range ordering (ERO). In the XRPD patterning of over-stoichiometric arsenoselenides, the FSDP-related peak halo observed in the range of ~15–22° 2*θ* reflects correlations between some polyhedron-like thioarsenide-type As_4_Se_n_ entities, while the SSDP-related halo in the range of ~28–33° 2*θ* indicates the orientational specificity of these polyhedra, attributed to second-order atomic pair correlations that are in close proximity to the mean interatomic spacing [21]. The shortest interatomic separation (~2.1–2.3 Å) results in the third diffraction peak (TDP) at ~(50–60)° 2*θ* (*Q*_3_~3.3–4.0 Å^−1^), insensitive to modification effects in glass [21,22].

STOE WinXPOW 3.03 [23] and PowderCell 2.4 [24] software were used to study the arrangement of diffuse peak halos in the XRPD patterns following normalization with respect to the maximum diffuse peak halo (which was the SSDP in the current case of arsenoselenides [11]). To clarify the fine details in the peak halo’s shape and positioning for arsenoselenide specimens, which essentially differ by their pre-history, we equilibrated the minimum level in the background of the collected XRPD patterns just before the SSDP-related peak halo. The uncertainties in the peak halo position (2*θ*) and full width at half maximum (FWHM) were less than ±0.05° 2*θ*; the scattering vector *Q* and width Δ*Q* were defined as (4π/*λ*)·sin*θ* and (4π/*λ*)·sin(FWHM/2), respectively. The characteristic distance *R* and correlation length *L* were defined as 2π/*Q* and 2π/Δ*Q*. As part of the revised microcrystalline model [12], the arrangement of diffuse peak halos of amorphous alloys was also treated as arising from diffraction of coordination spheres, i.e., the shortest interatomic distances like in randomly packed multiparticulate systems [25], where the XRPD is governed by the known Ehrenfest relation (2*d_s_*·sin*θ* = 1.23·λ) [26]. Notably, the error bars in the above linear parameters (*R*, *L*, *d_s_*) did not exceed ±0.1 Å.

The microstructure peculiarities of the examined As_4_Se_2_ specimens were also identified by Horiba Xplora micro-RS spectroscopy (Kyoto, Japan). The CW 785 nm laser of 90 mW output power was employed for excitation, with the 10% power option being used to avoid photostructural effects. Other measurement options were as follows: ×100 objective, 1800 L/mm grating, 500 μm hole, 50 μm slit, ~2 cm^−1^ spectral resolution, and ~2 μm spatial resolution. The number of spectral acquisitions was optimized relative to the surface area of each sample to ensure reproducibility of micro-Raman spectra analyzed with Horiba LabSpec. Comparative evaluation was conducted through normalization of specific spectral regions. Raman-active vibrational modes were assigned based on established reference data for chalcogenide materials [27,28,29,30,31,32,33,34].

Thermoanalytical heat-transfer phenomena in the examined As_4_Se_2_ alloy subjected to nanomilling were investigated by employing the DSC-TOPEM^®^ multifrequency temperature-modulated method [35,36,37,38,39,40,41,42] with a DSC-1 calorimeter (Mettler-Toledo, Greifensee, Switzerland) equipped with an FRS5+ sensor and a TC100 intracooler (Huber, Offenburg, Germany). The obtained data were processed by the STAR^e^ ver.13a software. Multi-point calibration was performed with a set of standard probes (n-Octane, Hg, In, Zn). The sample of interest (15–25 mg) was encapsulated in sealed Al pans in a N_2_ atmosphere, scanned at a 1.0 K·min^−1^ rate, and stochastically modulated in 0.75 K pulses between 15 s and 40 s.

Methodologically, the calorimetric measurements were arranged in two cycles, including subsequent heating–cooling runs within the (0–250) °C range. During the first heating run, when the calorimeter reached the maximum temperature of 250 °C, the cooling run towards 0 °C was started immediately under a constant rate approaching −5 K·min^−1^. After the completion of this heating–cooling cycle, the sample was kept at 0 °C for about 15 min, and then a second heating run was started under conditions identical to those in the first run. The processing of the collected data was adjusted using a sapphire reference spectrum, with the calculation window set to a width of 60 s and a shift interval of 1 s. In case of reference PVP probes, the measurements were performed under a preliminary temperature drop from 25 °C to −60 °C and then continued within subsequent heating–cooling runs (1 K/min) between −60 °C and 260 °C. Mathematical processing of the data was carried out using a calculation window with a width of approximately 120 s and a shift increment of 2 s.

The heat-transfer phenomena in the examined specimens of As_4_Se_2_ alloy were parameterized through DSC-TOPEM profiles presenting temperature variation of non-reversing (HF_nrev_) and reversing (HF_rev_) heat flow in the first and second heating runs [35,36]. The reversing thermal-alteration effects due to second-order transitions in thermodynamically unstable substances were described by heat capacity variations (ΔCp), allowing the determination of onset (*T*_g_^onset^) and mid-point (*T*_g_^mid^) glass transition temperatures. The non-reversing effects due to thermal relaxation towards the equilibrium state were defined by the specific enthalpy difference (ΔH) defined below the HF_nrev_(T) curve [35,36,37,38,39,40,41,42].

## 3. Results and Discussion

### 3.1. Medium-Range Structure and Microstructure Response in Nanostructured As_4_Se_2_ Alloy

**Figure 1:**
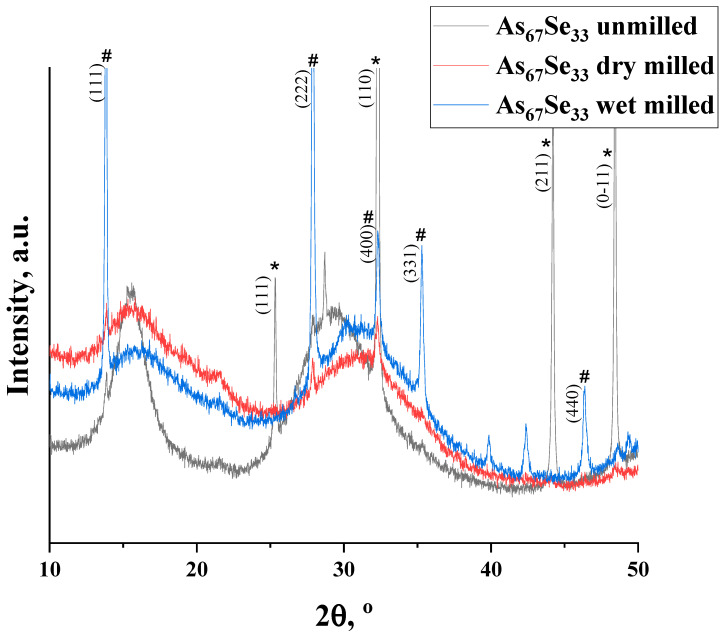
XRPD patterns in melt-quenched As_4_Se_2_ alloy before (black curve) and after nanomilling in dry (red curve) and dry–wet mode (blue curve), showing normalized profiles of the FSDP-SSDP-related diffuse peak halos overlapped with the most pronounced Bragg-diffraction reflexes originating from the rhombohedral As phase (denoted by asterisk symbol (*) with respect to JCPDS No. 72-1048 [16,17]) and cubic arsenolite As_2_O_3_ phase (denoted by hash symbol (#) with respect to JCPDS No. 36-1490 [18]).

The nanomilling-induced alteration in the medium-range structure of the examined alloy can be parameterized across hierarchical levels of the IRO and ERO corresponding to the FSDP- and SSDP-related diffuse peak halos in their XRPD patterning normalized to the SSDP-related peak halo in nanomilled samples (see Figure 1); the parameters of the medium-range structure extracted from these XRPD patterns are summarized in Table 1.

The XRPD patterns of the As_4_Se_2_ specimens reveal a three-peak-halo structure (composed of three distinct diffuse peak halos typical for vitreous chalcogenides, the FSDP, SSDP, and TDP [21]), superimposed with sharp–broadened Bragg-diffraction reflexes from some crystalline inclusions ascribed to elemental As (corresponding to rhombohedral grey α-As [15,16]) and its oxide (corresponding to cubic arsenolite As_2_O_3_ [18]). In the melt-quenched alloy (unmilled), the elemental As prevails over As_2_O_3_ and the amorphous arsenoselenide phase due to sharp reflexes of this phase superimposed on the FSDP-SSDP-related diffuse peak halos (see Figure 1, black curve). After dry nanomilling in an Ar atmosphere, the FSDP-SSDP-related peak halos (enhanced in decaying sequence in their intensities due to residual stress generated under mechanical grinding [12]) prevail over broadened ‘crystalline’ reflexes, confirming preference of reamorphization transitions in this alloy (see Figure 1, red curve). Additional milling in the water solution of PVP advances subsequent As extraction and oxidation, this effect being revealed due to enhanced reflexes of the both As and As_2_O_3_ phases superimposed on suppressed diffuse peak halos attaining some irregularity due to domination of the SSDP-related peak halo signalizing a more stable state of the wet-milled specimen (see Figure 1, blue curve) [11,12]. The crystallization processes in the examined arsenoselenides are well revealed due to the normalized profile of the FSDP-SSDP-related diffuse peak halos overlapped with sharply enhanced Bragg-diffraction reflexes from planes (111) at 25.36° 2*θ* (*d* = 3.5149 Å, *I* = 13%), (110) at 32.32° 2*θ* (*d* = 2.7710 Å, *I* = 100%), (211) at 44.20° 2*θ* (*d* = 2.0491 Å, *I* = 29.9%), and (0-11) at 48.40° 2*θ* (*d* = 1.8805 Å, *I* = 33.3%) in rhombohedral As (JCPDS No. 72-1048 [16,17]), as well as (111) at 13.86° 2*θ* (*d* = 6.390 Å, *I* = 63%), (222) at 27.90° 2*θ* (*d* = 3.195 Å, *I* = 100%), (400) at 32.33° 2*θ* (*d* = 2.769 Å, *I* = 27%), (331) at 35.32° 2*θ* (*d* = 2.541Å, *I* = 38%), and (440) at 46.4° 2*θ* (*d* = 1.9578 Å, *I* = 39.4%) in cubic arsenolite As_2_O_3_ (JCPDS No. 36-1490 [18]).

The position of the FSDP-related diffuse peak halo in melt-quenched (unmilled) As_4_Se_2_ alloy at 15.48° 2*θ* (*R*~5.71 Å, *d_s_* = 7.0 Å, see Table 1) exhibits good agreement with the positioning of the most intensive inter-planar (Bragg diffraction) correlations in crystalline arsenoselenides (As_2_Se_3_, As_4_Se_4_, and As_4_Se_3_) approaching *R*~5.64 Å [11,12]. This finding means an essential contribution to the FSDP from the Ehrenfest diffraction ascribed to interatomic and/or inter-molecular correlations within thioarsenide-type As_4_Se_n_ (6 < *n* < 0) molecules and their low-order network-forming derivatives with average distance *d_s_* beyond ~7 Å [12]. Under milling, the destruction of As_4_Se_n_ molecules and reincorporation of their remnants into a network composes a reamorphization (molecular-to-network) trend resulting in an increase in the FSDP position *Q* and width ∆*Q* corresponding to the fragmentation impact on the correlation length of the FSDP-responsible entities (*L*). The disruption of IRO due to the weakening of the FSDP-responsible peak halo is accompanied by the enhancement of ERO due to fragmentation of the SSDP-responsible entities, increasing the position and width of the SSDP-related peak halo (see Table 1). Thus, these transformations occur as an interplay between different levels of IRO and ERO in the alloy subjected to nanomilling [12].

In the melt-quenched As_4_Se_2_ alloy subjected to dry-nanomilling, the FSDP position slightly shifts towards higher *Q* values (see Figure 1, red curve), while the FSDP width ∆*Q* demonstrates a significant increase, testifying in favor of a very strong amorphization trend in this specimen. Additional wet milling leads to drastic broadening in the FSDP and SSDP width (Figure 1, blue curve), testifying in favor of an enhanced amorphization trend in this dry-nanomilled alloy. The remnants of crystalline structures associated with inter-molecular correlations with ~7 Å inter-centroid distances (contributing to the Ehrenfest diffraction patterning) are merely destroyed under wet milling, while those responsible for inter-planar correlations advance further to the XRPD patterning by the Bragg diffraction. Because of molecular-to-network transformations, the broadened and depressed diffuse peak halos in the wet-nanomilled specimens become more shifted towards higher *Q* values. Since the remnants of thioarsenide-type molecular entities degraded during grinding interact with oxygen, particularly in the PVP aqueous solution, the As_2_O_3_ phase is stabilized in the As_4_Se_2_ alloy under wet milling.

These findings are concomitant with the microstructure response to nanomilling-driven amorphization in As_4_Se_2_ alloy derived from the micro-RS spectroscopy analysis.

**Figure 2:**
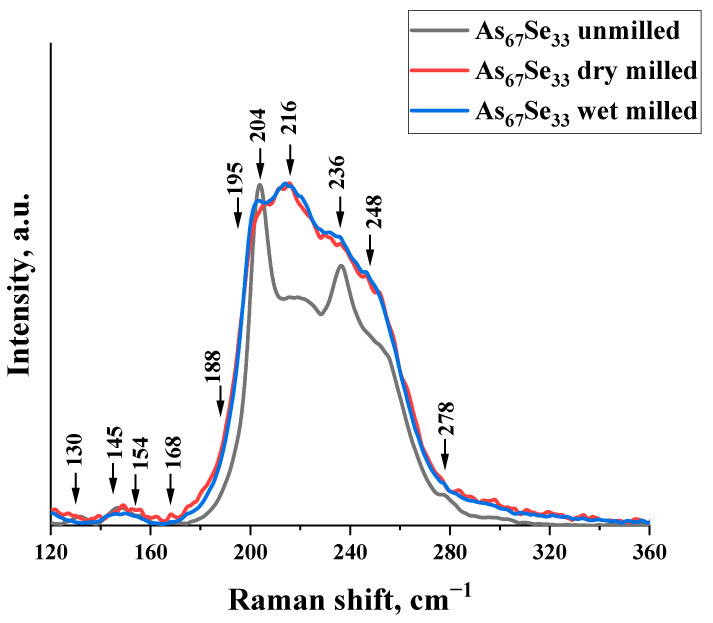
Normalized micro-RS spectra of the As_4_Se_2_ alloy in unmilled (black curve), dry-nanomilled (red curve), and dry–wet-nanomilled (blue curve) states. See the text for more details on the assignment of the most prominent features in the collected micro-RS spectra.

Thus, in the normalized micro-RS spectrum of melt-quenched As_4_Se_2_ alloy, a few low-frequency (~130, 145, 154, 168, and 188 cm^−1^) and high-frequency (~195, 204, 216, 236, 248, and 278 cm^−1^) bands are well defined (see Figure 2, black curve). The broad band at 210–230 cm^−1^ is ascribed to overlapped bond-stretching modes of AsSe_3_ pyramids [9,27] and thioarsenide-type molecules such as As_4_Se_4_ (peaks at ~225 and 240 cm^−1^ and shoulder near 255 cm^−1^ [9,28,29,30]), As_4_Se_3_ (peaks at ~195, 205, 225, 240, and 255 cm^−1^ and shoulder near 280 cm^−1^ [28]), and As_4_ (peak at ~200–210 cm^−1^ [9,35,36]). The low-frequency bands are assigned to bond-bending modes in thioarsenide molecules such as As_4_Se_4_ (~145, 160, and 170 cm^−1^ [32,37,38]) and As_4_Se_3_ (~110, 135, 150, 160, and 170 cm^−1^ [28,33]). The appearance of As_4_ molecules in this alloy is expected due to the RS-active band at 204 cm^−1^, while As_4_Se_4_ and As4Se3 molecules are evidenced by a distinct band at 236 cm^−1^, supplemented by shoulders near ~255 cm^−1^ and ~278 cm^−1^. The spectral region between these bands corresponds to vibrational modes of AsSe_3_ pyramids incorporated in the arsenoselenide network. As a result, the micro-RS spectrum of the melt-quenched As_4_Se_2_ alloy (depicted by a black solid curve in Figure 2) has a characteristic double-peak shape with distinct and strong maxima positioned near ~200 and ~240 cm^−1^ accompanied by hump at ~(210–230) cm^−1^ and relatively weak shoulders revealed near ~255 and ~280 cm^−1^.

These fine features are also well observable in the micro-RS spectra of dry and dry–wet-nanomilled specimens (see Figure 2, red and blue curves), but they are essentially broadened and obscured as compared with the micro-RS spectrum of the unmilled As_4_Se_2_ alloy. This finding is in line with the most generalized nanostructurization-driven amorphization trends in over-stoichiometric arsenoselenides [12]. Molecular-to-network transformations occur in these substances owing to the destruction of thioarsenide-type molecular entities, proceeding with incorporation of their remnants in a newly polymerized arsenoselenide network undergoing polyamorphic (amorphous-I-to-amorphous-II) transitions [11,12].

### 3.2. Calorimetric Heat-Transfer Response in Nanostructured As_4_Se_2_ Alloy

**Figure 3:**
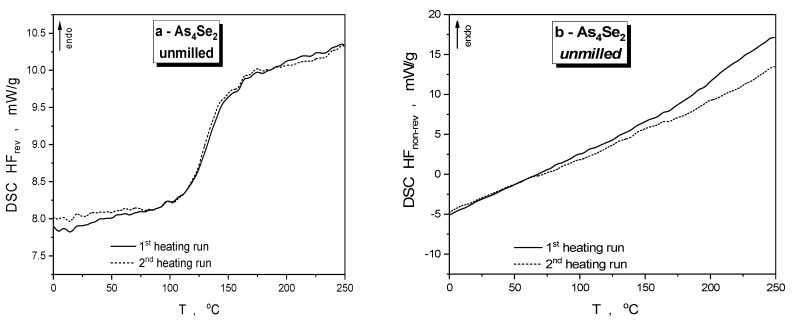

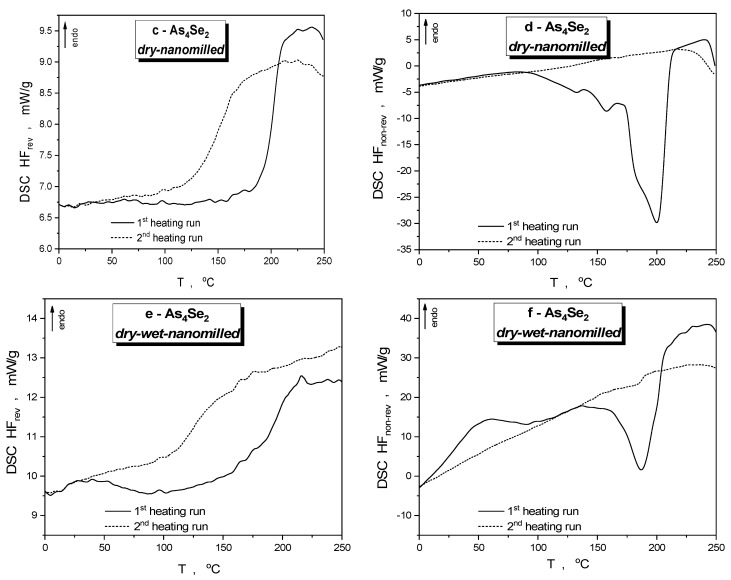
DSC-TOPEM profiles of specimens of As_4_Se_2_ alloy, unmilled (**a**,**b**) and after nanomilling in dry (**c**,**d**) and dry–wet mode (**e**,**f**), showing reversing HF_rev_ (**a**,**c**,**e**) and non-reversing HF_nrev_ (**b**,**d**,**f**) heat flow variations in the first (solid curve) and second heating run (dotted curve).

The specimens of As_4_Se_2_ alloy affected by defects generated under high-energy nanomilling become notably stressed, enhancing the calorimetric heat-transfer response as compared with the as-prepared (unmilled) specimens derived by melt-quenching. The DSC-TOPEM profiles depicted in Figure 3a,b specify temperature variations of reversing (HF_rev_) and non-reversing (HF_nrev_) heat flow collected in the first and second heating runs in this alloy, and respective parameters of the DSC profiles are gathered in Table 2.

During the initial heating cycle, the primary endothermic thermal transformation event in the examined amorphous substances is the glass transition [39]. For the unmilled As_4_Se_2_ specimen, the HF_rev_ curve (see Figure 3a) shows characteristic stepwise jump resulting in heat capacity variation Δ*C*_p_~0.10 J·g^−1^·K^−1^ and onset glass transition temperature *T*_g_^onset^~116.6 °C (in good agreement with the known data [39]), while the HF_nrev_ curve in Figure 3b demonstrates a smoothly growing endothermic tendency without any distinct peaks. It seems reasonable that despite its heterogeneous glassy–crystalline conformation, this alloy is in a quite relaxed state characteristic of long-term-aged or thermally annealed arsenoselenides [10,11], as could also be inferred from the nearly invariant steplike jump in the HF_rev_ and small high-temperature decrease in the HF_nrev_ detected in the second heating run in the DSC-TOPEM profiles shown by dotted lines in Figure 3a,b. Such specificity of the heat-transfer phenomena is explained by low-polymerized molecular-network conformations in melt-quenched As_4_Se_2_ alloy enriched in thioarsenide-type As_4_Se_n_ molecules and their iso-compositional network-forming derivatives [12].

Calorimetric heat-transfer responses in the examined As_4_Se_2_ alloy subjected to nanomilling in a dry mode are specified by the DSC-TOPEM profiles reproduced in Figure 3c,d, showing reversing (HF_rev_) and non-reversing (HF_nrev_) heat flow variations in the first and second heating runs. Because of the nanomilling-driven depression in molecularity [12], the glass transition temperature of this dry-nanomilled specimen *T*_g_^onset^ gradually rises to 191.7 °C with Δ*C*_p_~0.16 J·g^−1^·K^−1^ (see Table 2), thereby approaching this parameter in stoichiometric As_2_Se_3_ possessing a 2D layer-type network structure [8,40]. The abnormous network-enhanced increase in the glass transition temperature of this As_4_Se_2_ alloy possessing a mixed molecular-network conformation can be interpreted as a signature of nanomilling-driven reamorphization transitions typical for over-stoichiometric As-bearing arsenoselenides derived by conventional melt-quenching [11,12]. The temperature variation of non-reversing (HF_nrev_) heat flow in the dry-nanomilled As_4_Se_2_ specimen in the first heating run (reproduced by solid line in Figure 3d) exhibits a strong exothermic peak near ~(190–195) °C supplemented by a few weak features at ~130, ~155, and ~180 °C. At the basis of preliminary microstructure research confirming amorphization transformations in this alloy independently of crystal inclusions [12], it could be speculated that this exothermic multipeak in HF_nrev_ heat flow (accompanied by specific enthalpy difference ΔH~−73.4 J·g^−1^, see Table 2) may result from diversity in the stress generated by high-energy mechanical milling in a structurally inhomogeneous arsenoselenide alloy composed of thioarsenide As_4_Se_n_ molecules and their network derivatives. After the first cooling run from 250 °C to 0 °C, the dry-nanomilled As_4_Se_2_ specimen is rejuvenated, returning closer towards the initial stress-free state with only a small residual component. Thus, the glass transition temperature in this dry-nanomilled specimen derived from the HF_rev_(T) curve (depicted by dotted line in Figure 3c) is partially recovered by thermal treatment within the first heating–cooling cycle, resulting in *T*_g_^onset^~136.7 °C (see Table 2).

Temperature variations of the reversing (HF_rev_) and non-reversing (HF_nrev_) heat flows collected in the first and second heating runs in the dry-nanomilled As_4_Se_2_ alloy additionally subjected to wet milling (in the 0.5% PVP water solution) are depicted by the DSC-TOPEM profiles reproduced in Figure 3e,f. The essential difference of this wet-nanomilling-driven calorimetric response from that caused by dry-nanomilling is obvious even from visual inspection of these heat flow variations. In PVP-capped nanocomposite fabricated by wet milling (that is As_4_Se_2_/PVP nanocomposite), a broad endotherm peaking at ~40 °C and stretching to almost ~100 °C appears in the first heating run in the HF_rev_(T) dependence before a *T*_g_^onset^-related steplike jump at ~179.4 °C associated with Δ*C*_p_~0.13 J·g^−1^·K^−1^ (see Table 2). In non-reversing heat flow determination (see Figure 3f), this event is revealed as a smoothly growing endotherm in the (0–100) °C range with ΔH~25.6 J·g^−1^ followed by a strong asymmetric exotherm in the (130–200) °C range peaking at ~180 °C with ΔH~53.4 J·g^−1^ (see Table 2). After the first cooling run, the wet-milled specimen is rejuvenated, returning almost completely to its initial state free of residual mechanical stress. Thus, the glass transition temperature in this As_4_Se_2_/PVP nanocomposite derived from HF_rev_(T) dependence (see Figure 3e) returns back to *T*_g_^onset^~112.4 °C with heat capacity variation Δ*C*_p_~0.11 J·g^−1^·K^−1^ (see Table 2), while the HF_nrev_ curve reproduced in Figure 3f demonstrates a smoothly growing endothermic tendency without any notable peaks.

**Figure 4:**
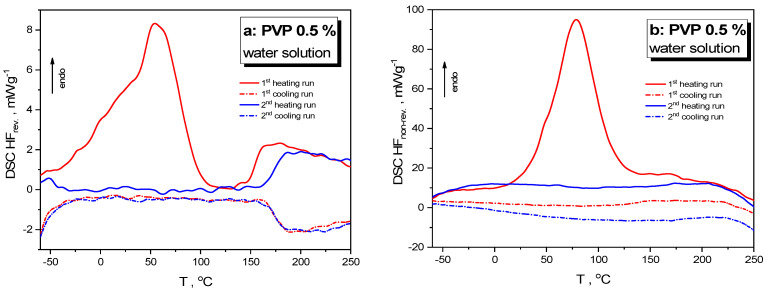
DSC-TOPEM profiles of the pelletized samples of wet-nanomilled PVP 0.5% water solution showing variations of reversing HF_rev_ (**a**) and non-reversing HF_nrev_ heat flow (**b**) in the respective runs of the first heating (red solid curve) followed by cooling (red dotted curve) and the second heating (blue solid curve) followed by cooling (blue dotted curve). See the text for more details.

To shed more light on calorimetric heat-transfer responses in the As_4_Se_2_ alloy nanostructured under wet milling, similar phenomena were examined in the reference sample of the PVP water solution (subjected to wet milling under the same conditions). The DSC-TOPEM profiles for such pelletized reference samples showing reversing (HF_rev_) and non-reversing (HF_nrev_) heat flow variations in the heating–cooling runs starting from −60 °C are reproduced in Figure 4a and Figure 4b, respectively.

In the first heating run, the HF_rev_(T) dependence (red solid curve, Figure 4a) shows broad multi-featured endotherm within the (−60–100) °C range peaking at ~50 °C and a distinct steplike jump related to glass transition at ~155 °C, while the HF_nrev_(T) dependence (red solid curve, see Figure 4b) shows a strong endotherm within the (20–130) °C domain peaking at ~75 °C. In the second heating run (after rejuvenation during the first heating–cooling cycle), the endotherm in the HF_rev_(T) dependence within the (−60–100) °C range disappears, and the glass transition jump shifts to almost ~170 °C, while the endotherm peak in the HF_nrev_(T) dependence disappears completely. The difference in the glass transition temperature defined by a jump in the HF_rev_(T) dependence approaching ~15 °C can be completely attributed to the known effect of nanostructurization-driven depression in the glass transition temperature [6].

The heat-transfer events in organic-based hydrogel systems are known to be defined by water, which behaves as a plasticiser in amorphous materials like PVP, with the water molecules being bonded to carbonyl groups as well as to the C–N sites in the PVP molecules [41,42,43,44]. Under a small water concentration (at the level of ~0.5%), the water molecules are distributed more or less uniformly throughout the PVP, but they become notably heterogeneous at higher content, with clusters of molecules occupying different channels between polymer chains [41,42].

A similar effect can be driven by high-energy mechanical milling. It could be reasonably anticipated that the smoothly growing endotherm revealed in temperature variation of non-reversing HF_nrev_ heat flow in the As_4_Se_2_/PVP nanocomposite within the (0–100) °C range (see Figure 3f) results from multistage water elimination from the PVP under nanomilling. Because of high-energy grinding, structurally heterogeneous domains with different water contents appear in the As_4_Se_2_/PVP nanocomposite fabricated by wet nanomilling, leading to differentiation of the collected DSC-TOPEM profiles from this specimen.

By finalizing, the poorly polymerized molecular-network conformations in over-stoichiometric arsenoselenides such as tetra-arsenic biselenide As_4_Se_2_ alloy enriched with a high content of thioarsenide molecules and their network derivatives result in a gradually enhanced effect of nanostructurization, driving the glass transition temperature increase. This effect is well pronounced in the As_4_Se_2_ alloy subjected to dry nanomilling, while it is partly counterbalanced in this alloy subjected to wet milling in the PVP water solution, accompanied by the stabilization of the As_4_Se_2_/PVP nanocomposite.

In the perspective of contemporary chalcogenide mechanochemistry research (see, e.g., [45,46,47,48]), this result underlines an essential role of dry- and wet-nanomilling processing technologies in achieving optimized multifunctional responses in disordered substances governed by their molecular-network microstructure.

## 4. Conclusions

Nanomilling-driven effects on polyamorphic transitions are examined in As_67_Se_33_ alloy, equivalent to tetra-arsenic biselenide As_4_Se_2_, just at the boundary of the glass-forming region in the As-Se system. These alloys were subjected to thermophysical characterization by the multifrequency temperature-modulated DSC-TOPEM^®^ method and microstructure characterization by X-ray powder diffraction and micro-Raman spectroscopy analysis.

As shown by XRPD analysis, this alloy reveals a glassy–crystalline nature due to inclusions of rhombohedral As and cubic As_2_O_3_ (arsenolite) phases in the amorphous As-Se network, and these phases especially grew after wet nanomilling in a PVP (polyvinylpyrrolidone) water solution. At the medium-range structure level, the nanostructurization-driven changes in this alloy are revealed as the disruption of intermediate-range ordering accompanied by the enhancement of extended-range ordering. The most generalized molecular-to-network amorphization trend in this As_4_Se_2_ alloy subjected to nanomilling is confirmed by the microstructure response revealed in the broadened and obscured features in the collected micro-Raman scattering spectra of dry- and wet-nanomilled samples.

Thermophysical heat-transfer phenomena in As_4_Se_2_ alloy are defined by molecular-to-network polyamorphic transformations activated under nanomilling in dry and dry–wet modes. The domination of thioarsenide-type As_4_Se_n_ molecular entities in this alloy results in an abnormal nanomilling-driven network-enhanced glass transition temperature increase. The nanostructured As_4_Se_2_ alloy becomes notably stressed due to the destruction of thioarsenide molecules and incorporation of their remnants into a newly polymerized arsenoselenide network. This effect is well pronounced in the alloy subjected to dry nanomilling, while it is partly counterbalanced in preliminary dry-nanomilled specimens subjected to wet milling in a PVP water solution, accompanied by the stabilization of the As_4_Se_2_/PVP nanocomposite.

## Figures and Tables

**Table 1:** Characterization of the FSDP- and SSDP-related diffuse peak halos in the XRPD patterning of As_4_Se_2_ alloy before and after milling.

Specimen State	Peak Halo	Peak Halo’s Parameterization						
		2*θ*, °	FWHM, °	*Q*, Å^−1^	∆*Q*, Å^−1^	*R,* Å	*L,* Å	*d_s_*, Å
unmilled	FSDP	15.48(1)	2.34(1)	1.098	0.166	5.71	37.8	7.0
	SSDP	29.99(1)	5.91(1)	2.110	0.420	2.98	14.9	3.7
dry-nanomilled	FSDP	15.73(3)	3.77(6)	1.116	0.268	5.63	23.4	6.9
	SSDP	31.56(1)	6.49(3)	2.216	0.455	2.83	13.8	3.5
dry–wet-nanomilled	FSDP	15.89(2)	5.08(4)	1.127	0.362	5.57	17.4	6.9
	SSDP	31.36(1)	7.13(2)	2.204	0.507	2.85	12.4	3.5

**Table 2:** Parameterization of DSC profiles obtained from reversing HF_rev_ and non-reversing HF_nrev_ heat flow in melt-quenched As_4_Se_2_ alloy before and after nanomilling.

Stage of DSC-TOPEM Profile	State of As_4_Se_2_ Alloy	Calculated from HF_rev_			Calculated from HF_nrev_
		Glass Transition Temperature		Heat Capacity Variation	Specific Enthalpy Difference
		*T*_g_^onset^, °C	*T*_g_^mid^, °C	Δ*C*_p_, J·g^−1^K^−1^	Δ*H*, J·g^−1^
First heating run	unmilled	116.6	131.2	0.10	-
	dry-nanomilled	191.7	201.2	0.16	−73.4 (exotherm multipeak)
	dry–wet-nanomilled	179.4	192.7	0.13	25.6 (endo); −53.4 (exo)
Second heating run	unmilled	115.5	129.3	0.10	-
	dry-nanomilled	136.7	148.8	0.10	-
	dry–wet-nanomilled	112.4	129.1	0.11	-

## Data Availability

The original contributions presented in this study are included in the article. Further inquiries can be directed to the corresponding author.
